# Ultrasound for Volume Assessment in Patients with Shock: Effectiveness of an Educational Intervention for Fourth-year Medical Students

**DOI:** 10.7759/cureus.2129

**Published:** 2018-01-30

**Authors:** Paul Kukulski, Michael Ward, Keme Carter

**Affiliations:** 1 Emergency Medicine, The University of Chicago Medicine; 2 Emergency Medicine, University of Wisconsin

**Keywords:** emergency medicine, education, ultrasound, shock, sepsis, clerkship, echocardiography, inferior vena cava, didactic, curriculum

## Abstract

Objective

Shock is a common emergency condition with high morbidity and mortality, and judicious fluid resuscitation can significantly affect outcomes. The use of a bedside echocardiogram and evaluation of the inferior vena cava (IVC) via ultrasound (US) for collapsibility can predict volume status. Additionally, the Association of American Medical Colleges (AAMC) Entrustable Professional Activities (EPA) 10 states that residents need to be able to address a patient with a critical illness, including hypotension, on Day 1 of residency. Existing literature revealed no published curriculum to teach medical students these skills. We aimed to determine the effectiveness of an educational intervention to teach fourth-year medical students how to utilize IVC US measurement and echocardiography to assist in volume assessment of patients presenting with shock.

Methods

Students participated in an hour session on the first day of the emergency medicine (EM) clerkship. Didactic effectiveness was evaluated by comparing results on a pre-test and post-test. The test was administered to residents and attendings during the first week of the academic year to gain evidence for content validity. Students also responded to a survey to evaluate learner satisfaction.

Results

The average score on the validation test was 68.4% (standard deviation (SD): 21.6%, number (n) = 38) for residents and attendings, and 47.4% (SD: 19.4, n = 13) for interns. Students scored an average of 45.6% (SD: 23.6, n = 83) on the pre-test and 66.4% (SD: 22.1 n = 72) on the post-test, p < 0.01 (degrees of freedom (df) = 153, t = 5.7), Cohen's d = 0.92. The satisfaction survey showed 97.6% of students felt the session was worthwhile, 96.4% would recommend it to other students, and 83.1% felt it taught new information.

Conclusion

These results show that the educational intervention provides a significant increase in knowledge regarding volume assessment and the use of echocardiogram and IVC US. Additionally, students rated the course highly and felt that it provided information not otherwise taught in medical school. This curriculum addresses the AAMC EPA 10, as it increases students’ readiness to address hypotension and could add significant value to the medical school curriculum.

## Introduction

Shock is a common, high-risk emergent condition, and accurate volume assessment, informing resuscitation strategy, can significantly affect outcomes [1]. This is clearly illustrated in the case of septic shock, a leading cause of death in emergency department patients [1]. The most recent evidence shows that early goal-directed therapy (EGDT) does not improve mortality over “usual care” but aggressive fluid resuscitation remains a mainstay of treatment [2-4]. Aggressive fluid resuscitation requires identifying patients that will be fluid-responsive, as an inappropriate fluid administration may have deleterious effects [5]. Central venous pressure, a core component of EGDT, has not been shown to correlate with fluid responsiveness [6]. However, dynamic inferior vena cava (IVC) measurement via ultrasound (US) has been shown to predict an increase in cardiac output in response to a fluid bolus in mechanically ventilated [7-11] and spontaneously breathing patients [12-13].

Residents across all specialties are frequently the first and primary providers of patients in shock but may lack the necessary diagnostic skills. Multiple studies have shown that students and junior level residents have inadequate knowledge regarding the most appropriate sepsis care [14-17]. Increased education helps, as previous studies have shown that hands-on simulation improves students’ and residents’ knowledge of how to manage shock [18-20]. Furthermore, broad curricula aimed at physicians of all levels of training have been shown to improve mortality from shock [21]. Additionally, previous general US curricula have shown that short US courses are feasible and effective [22-25]. Parks et al. observed that students taught hands-on US for diagnosis of shock, including respiratory variation in IVC diameter and contractility on bedside echo, improved the students’ diagnostic accuracy of fluid responsiveness [26].

Finally, the Entrustable Professional Activities (EPA) for Entering Residency, set forth by the Association of American Medical Colleges (AAMC), delineates the minimum abilities required of a resident on the first day of training [27]. Activity 10 states that an intern must be able to recognize critical illness and implement a plan of care, including hypotension [27]. An education intervention utilizing US to assist in assessing volume status, given to all medical students prior to graduation, may help achieve the goal of taking care of patients in shock on the first day of residency.

To our knowledge, no educational intervention exists that teaches the valuable US skills necessary for the modern treatment of shock and fits into a medical school curriculum so that all students in a class participate. We developed a 60-minute didactic involving a lecture and hands-on skills session given to all medical students during the required emergency medicine clerkship. We predicted that the intervention would both increase knowledge on this important topic and that students would report high levels of learner satisfaction.

## Materials and methods

Curriculum design

Fourth-year medical students participated in the didactic during the emergency medicine (EM) clerkship for the academic year 2015-16. This group was chosen as the clerkship is required, and thus all graduating students would participate. The clerkship is one month long, enrolling between three and 13 students per month from July to April. Orientation to the clerkship occurs on the first day and includes six hours of didactic time, of which one hour was devoted to fluid management and US.

The intervention was composed of two parts: a 20-minute lecture portion and a 40-minute hands-on US skills session. The didactic included background on the types of shock, the importance of fluid resuscitation in sepsis, and the consequences of fluid overload; the topics were chosen by expert consensus. Students then participated in two interactive case discussions, which involved two different patients with near similar vitals and a vague history. One patient presented in septic shock while the other presented in cardiogenic shock. The students learned how US can be an important modality to differentiate between the two diagnoses with different management pathways. Finally, the students were taught how to obtain and interpret images of the IVC and heart using video examples. The video examples included an IVC, which had a diameter that varied with respiration and one that did not, and echocardiographic (ECG) images with a normal ejection fraction and another with a poor ejection fraction.

The US skill session consisted of the students breaking into two groups, each with a senior resident or attending. The students had supervised practice at obtaining images on human models of the IVC in one group and the heart in the other group and were asked to interpret their findings for the resident or attending. The students would switch groups halfway through the session so they would practice both IVC US and ECG. Each student was required to obtain each image at least once on the final assessment of their skills.

Curriculum evaluation

Kirkpatrick’s model for curriculum evaluation was used as a framework for the evaluation of the intervention, focusing on Levels 1 and 2 for this initial study [28].

Kirkpatrick Level 1 - reaction: Following the session, students were given a six-item written survey (Appendix 1) to assess learner satisfaction. Items were assessed on a five-point Likert scale.

Kirkpatrick Level 2 - learning: The students took a six-question multiple choice paper-based pre-test (Appendix 2) to establish baseline knowledge regarding sepsis, fluid resuscitation, IVC US, and bedside echocardiography prior to the training session. We gathered content validity evidence for the test both by administering it to EM attendings and residents and had it reviewed by the department’s ultrasound director.

At the end of the month-long clerkship, students were asked to complete an online post-test consisting of the same knowledge-based questions asked on the pre-test.

The primary outcome (Kirkpatrick Level 2) was the difference in scores between the students’ pre- and post-tests. We assessed the scores for a statistically significant difference using a two-tailed Student’s t-test. The effect size was calculated using Cohen’s d formula. The secondary outcome (Kirkpatrick Level 1) was learner satisfaction. Survey results were analyzed by taking the overall percentage of students who either “agree” or “strongly agree” with each statement. Statistical analysis was performed using Microsoft Excel® (Microsoft Corp., Redmond, WA).

The protocol was reviewed by the University of Chicago Institutional Review Board and considered exempt. Consent was obtained from all participants in this study.

## Results

The knowledge-based test was distributed to EM attendings, senior residents, and interns to gain validity evidence. Thirty-eight out of 49 (78% response rate) residents and attendings and 13/15 (87% response rate) interns completed the test. The residents and attendings scored an average of 68.4% (SD: 21.6, n = 38) on the six-question test. Incoming interns scored an average of 47.4% (SD: 19.4, n = 13).

**Figure 1:**
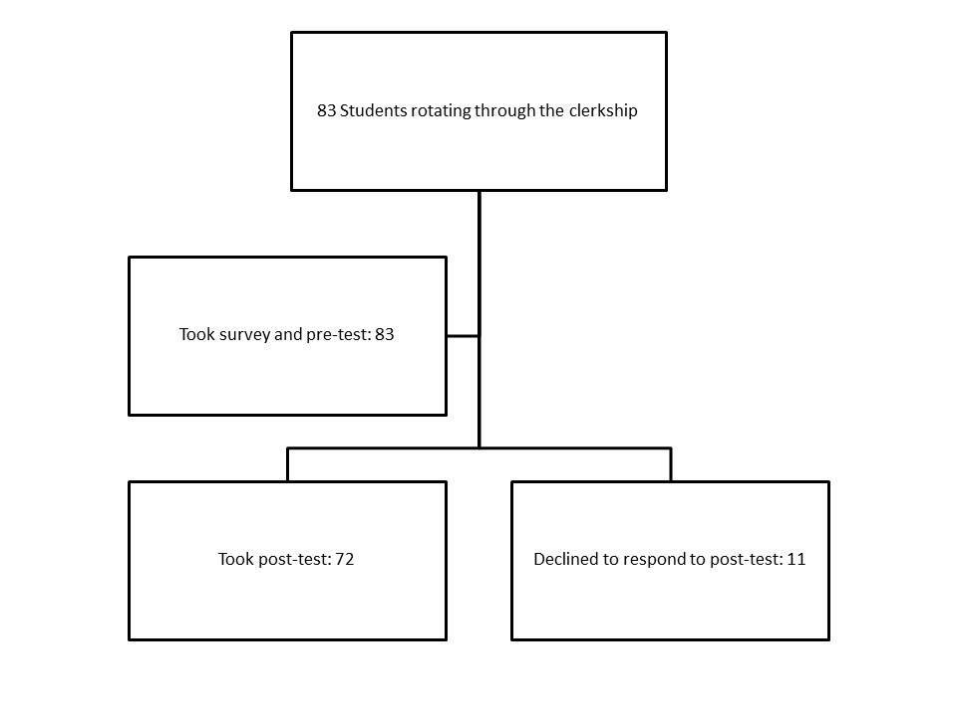
Flow of study subjects

Eighty-three students were enrolled in the clerkship during the 2015-2016 academic year. All were required to be present for the curriculum. All 83 students completed the paper-based pre-test and learner satisfaction survey. Seventy-two students completed the online post-test at the end of the clerkship, resulting in a response rate of 87% (Figure 1).

**Figure 2:**
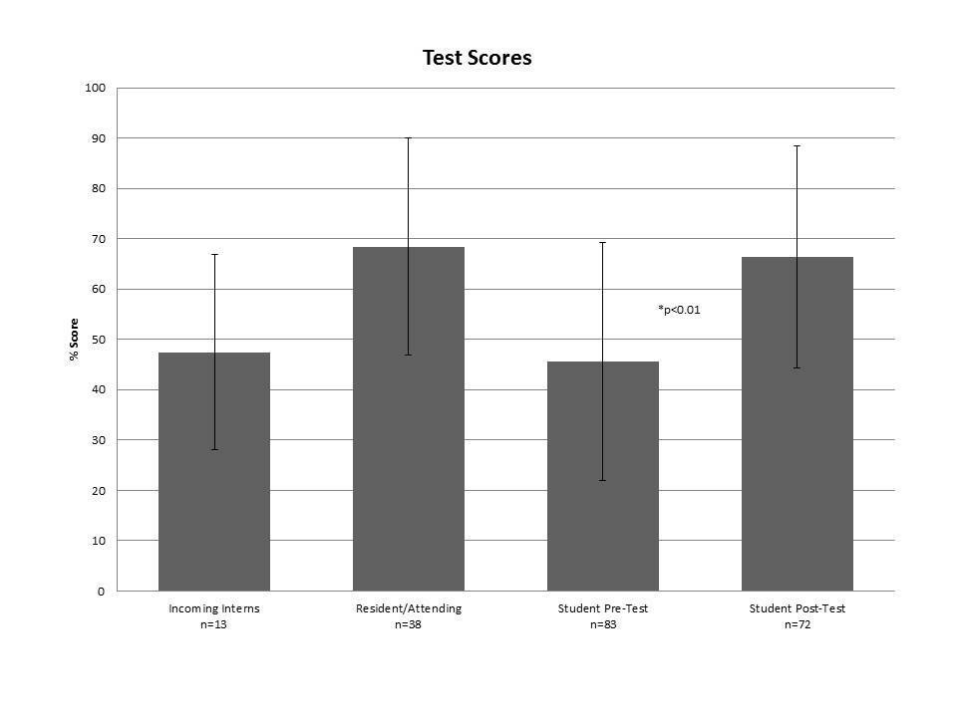
Primary outcome - knowledge test scores Scores represented as averages, bars indicate standard deviation. P-value represents comparision between student pre- and post-test scores

Prior to receiving the curriculum, students scored an average of 45.6% (SD 23.6, n = 83) on the knowledge test. At the end of the rotation, the average score on the test was 66.4% (SD 22.1, n = 72) with an absolute score increase of 20.8%, p < 0.01 (degrees of freedom (df) = 153, t = 5.7), Cohen's d = 0.92 (Figure 2).

**Figure 3:**
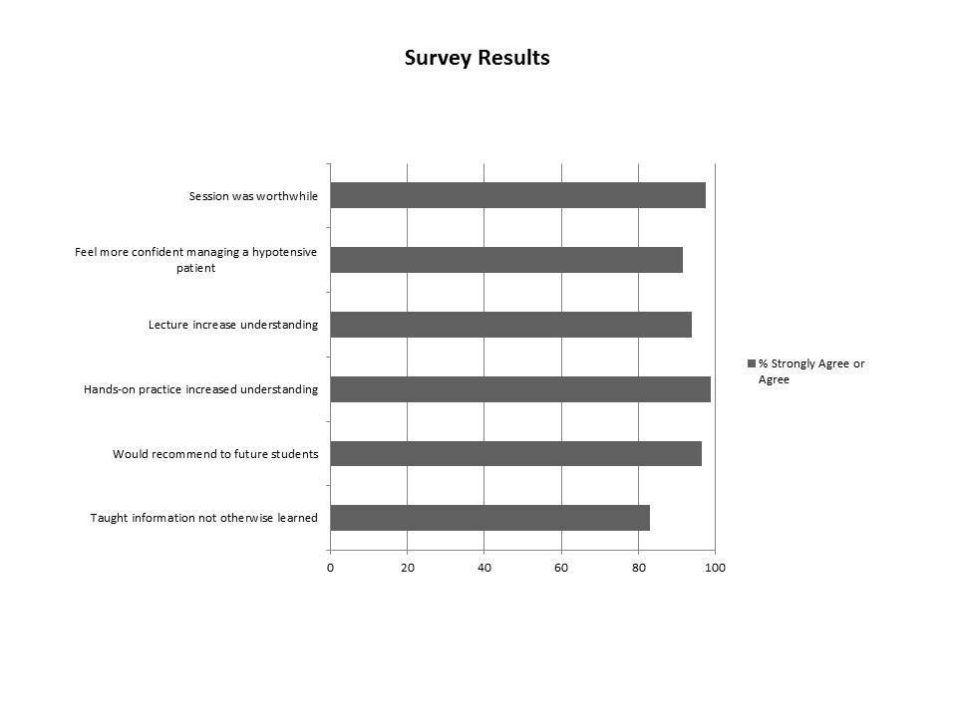
Secondary outcome - learner satisfaction survey results

Learner satisfaction survey results showed that 97.6% of students agreed or strongly agreed that “the fluid management session was worthwhile,” 96.4% of students agreed or strongly agreed that “I would recommend the session to further medical students,” and 83.1% of students agreed or strongly agreed that “Today’s session taught information not otherwise taught in the medical school curriculum” (Figure 3).

## Discussion

As studies continue to show that the use of US in the ED helps to improve the clinical assessment of patients presenting with shock [29-30], medical students will require formal instruction in its use. Through this curriculum, all fourth-year medical students at the study institution received instruction on how to utilize US to guide their resuscitation during the initial stages of treatment for a patient presenting with shock. Additionally, they received the opportunity to participate in a hands-on skills session to consolidate and practice what they had learned.

The average incoming intern score of 47.4% on our validation test (21.0% lower than the resident and attending score) provides further evidence that knowledge regarding the initial management of shock is lacking among interns beginning residency. This identifies a potential need for a curriculum aimed at fourth-year medical students prior to beginning residency. Given that most interns will take care of patients in shock and must be prepared to do so on the first day of residency, according to the EPA 10 [27], it is important that all students in medical school receive formal instruction on volume assessment prior to graduating.

Our didactic successfully provides a way to close this gap in knowledge. Scores on the pre- and post-test improved by 20.8% after the curriculum, which was a meaningfully significant increase in knowledge (a Cohen’s d effect size of 0.9 is considered large). This provides evidence that our didactic was an effective learning session.

Additionally, survey results indicated a very high score in the overall level of learner satisfaction. Nearly all (96-97%) of the students who went through the curriculum felt that it was a worthwhile addition and that future students should receive the curriculum. Importantly, a large number agreed that they learned information they would not otherwise have been taught in medical school, showing that the didactic may be a valuable addition to the curriculum.

To our knowledge, no other study has been published which incorporates the US evaluation of volume assessment into the medical school curriculum, such that all medical students receive the lesson. Our study showed that it is both feasible and worthwhile to include this didactic in the medical school curriculum.

This study does have limitations. First, we did not determine competency or retention of skill in the clinical setting – the third level of Kirkpatrick’s model for curriculum evaluation. Our didactic does, however, provide the foundation of knowledge for students to begin practicing this valuable skill (Level 2 of Kirkpatrick’s model). Secondly, since we were unable to compare results to a control group, although we showed the effectiveness and feasibility, we cannot say whether this intervention is superior to any other. Next, the participants in this study only included students from one medical school and may not represent the fund of knowledge or sentiments for students from other medical schools. We plan on addressing these three limitations with future studies. Finally, the survey determining learner satisfaction was anonymous but could be subject to a sponsor bias – there was no funding for this study; however, the students may have wanted to support the study’s results.

## Conclusions

According to the AAMC’s EPA 10, all residents should be able to recognize an unstable, hypotensive patient and implement a care plan on the first day of residency. Our didactic helps to achieve this goal by increasing students’ readiness to manage a patient with shock and could add significant value to their education.

## Appendices

Appendix 1: Learner Satisfaction Survey

Course Evaluation Survey

1)      Strongly disagree

2)      Disagree

3)      Neutral

4)      Agree

5)      Strongly agree

1.       The fluid management session was worthwhile______________________________________

2.       I feel more confident managing the fluid status on a hypotensive patient now than before the session:   

_____________________________________________________________________________

3.       The lecture portion helped increase my understanding _________________________________

4.       The ultrasound demonstration session helped increase my understanding _________________

5.       I would recommend this session to future medical students______________________________

6.       Today’s session taught information not otherwise taught in the medical school curriculum._____

Comments:

Appendix 2: Knowledge Assessment Test

Volume Assessment and Fluid Resuscitation Quiz

1)        Fluid resuscitation is LEAST likely to be helpful in which of the following types of shock?

a.       Distributive

b.       Cardiogenic

c.       Obstructive

d.       Hypovolemic

2)       According to more recent studies, which of the following is poorly predictive of volume status or fluid responsiveness?

a.        Pulse pressure variation

b.       Central venous pressure (CVP)

c.       Passive leg raise

d.       Respiratory variation in inferior vena cava (IVC) diameter

3)       What are the goals of resuscitation?

a.        Lactate clearance

b.       Mean arterial pressure (MAP) > 65

c.        Urine output 10 mL/kg/h

d.       A, B

e.       A, B, C

4)       Which of the following must you visualize on ultrasound to help differentiate the aorta from the IVC and to determine where you should estimate IVC respiratory collapse?

a.       The hepatic vein

b.       The liver

c.       The right atrium

d.       The spleen

e.       The superior vena cava (SVC)

5)       Using IVC respiratory variation, which statement below is correct?

a.       Underlying lung and cardiac disease do not impact respiratory variation of the IVC

b.       IVC respiratory variation of 50% in a mechanically ventilated patient and 20% in a spontaneously breathing patient both predict fluid responsiveness

c.        IVC respiratory variation of 20% is predictive of fluid responsiveness in both mechanically ventilated and spontaneously breathing patients

d.       IVC respiratory variation of 20% in a mechanically ventilated patient and 50% in a spontaneously breathing patient both predict fluid responsiveness

e.       When there is collapse of the IVC, it typically occurs during the inspiration phase in patients on mechanical ventilation and with inspiration in patients spontaneously breathing

6)       On bedside echo, what will the heart look like in the patient who is most likely to be fluid responsive?

a.        Minimal wall movement

b.        Dilated right ventricle

c.        Hyperdynamic wall movement

d.       Asymmetric wall movement

e.       Tachycardia
